# Impact of limited rest areas on truck driver crashes in Saskatchewan: a mixed-methods approach

**DOI:** 10.1186/s12889-020-09120-7

**Published:** 2020-06-19

**Authors:** Alexander M. Crizzle, Ryan Toxopeus, Jennifer Malkin

**Affiliations:** School of Public Health, University of Saskatchewan, 104 Clinic Place, Saskatoon, SK S7N 2Z4 Canada

**Keywords:** Long-haul truck drivers, Fatigue, Crashes, Truck stops, Rest areas

## Abstract

**Background:**

Long-haul truck drivers (LHTDs) suffer from long work hours often resulting in fatigue. Although several studies have reported that fatigue can contribute to crashes, no study has identified the location and patterns of fatigue-related crashes and solicited truck driver feedback on potential mitigation strategies. The purpose of this study is 1) to map the location of fatigue-related crashes and 2) examine the perceptions of truck drivers concerning fatigue-related crashes.

**Methods:**

Using databases from the Saskatchewan Government Insurance, information on LHTD demographics, crashes and their causes, as well as crash location was analyzed. All fatigue-related crashes were then documented and mapped. Additionally, we interviewed 67 LHTDs (mean age = 53.0 ± 12.9; range 23–89; 95% were men) asking questions about fatigue, access to truck stops/rest areas, and provided recommendations for improvement. All interviews were subsequently analyzed using thematic analyses.

**Results:**

On average, there were 20 fatigue-related crashes per year over the 10-year period. Fatigue-related crashes were common across Saskatchewan, however, there was a concentration of crashes along major roadways between major cities. There was a significant association between crashes with age and experience. Despite many LHTDs being fatigued, there was a lack of truck stops/rest areas along highway routes. LHTDs suggested having access to truck stops/rest areas 250–400 km apart with running water and washrooms available.

**Conclusions:**

Additional truck stops and rest areas are needed in Saskatchewan to ensure LHTDs have more opportunities for rest to reduce fatigue in general, as well as to reduce the risk of fatigue-related crashes.

## Background

Long-haul truck drivers (LHTDs) are exposed to long working hours and inconsistent sleep schedules, which can cause disruptions in both length and quality of sleep, resulting in over half of drivers reporting sleepiness while driving [1]. Additionally, fatigue may be exacerbated while driving in monotonous environments [2] such as the landscape seen in Central Canada. Multiple studies show that LHTDs have sleep-related problems ranging from 17 and 58% [3–5], with 32 to 32.8% of LHTDs reported making serious errors due to sleepiness [1, 5].

Fatigue in LHTDs is an occupational and public health concern given its association with crash risk. Fatigue can lead to inefficient processing of the driving environment [6], poorer executive function [7], more hard-braking events [8], and greater variance in steering and lane positioning [9]. Studies found that 6.9–7.5% of truck driver crashes are related to fatigue [1, 5]. Similarly, a U.S. study found that fatigue contributed to 4.2% of all LHTD crashes, however, those crashes accounted for 7.2% of non-life-threatening injuries, 13.1% of collision-related hospital admissions, and 16.5% of all fatal traffic crashes [10].

Although instances of crashes are generally low, it is estimated that the prevalence of fatigue-related accidents is higher than reported in research studies [11, 12]. LHTDs may be reluctant to report an accident caused by fatigue for employment and insurance purposes [13]. LHTD are subject to both acute and chronic (extreme) fatigue. Acute fatigue refers to general tiredness resulting from working long hours, poor sleep quality or driving monotonous routes, and without intervention, can become chronic/extreme fatigue after prolonged exposure [14]. Chronic fatigue results in poorer cognitive processing abilities further increasing the risk for crashes [15]. While there are programs available such as the North Fatigue Management Program that addresses fatigue in LHTDs [16], environmental changes are also needed to alleviate fatigue, such as increasing the number of safe parking locations [5].

Prior studies have examined truck parking at rest areas in Canada [17, 18]. One study found that 81% of 1788 truck drivers reported having difficulties finding parking in urban areas, which caused them to exceed their hours of service, and more than 88% mentioned that there were not enough rest areas, roadside pull-outs and turn-outs, or safe lanes to safely perform inspections. Additionally, amenities at current rest areas such as washrooms, drinking water, cellular phone service (in remote areas), food, showers, convenience stores and security (e.g., camera surveillance, fencing, lighting) were viewed as inadequate. The parking and rest stop shortage contributes to drivers exceeding their hours of service [17], as well as to driver fatigue and illegal parking [18], all of which are related to accidents. In short-haul truck drivers, difficulty finding parking was associated with a 3.7x increased risk of being in a crash [19]. However, none of these studies examined where crashes occurred or where truck stops and rest areas were needed.

Prior studies in industrial safety found that the risk of accidents increased after 2-h of continuous work. A 10-min break every two hours, however, reduced the risk of accidents to that of baseline [20]. Soccolich and colleagues [21] found that a 30-min break (non-driving periods) reduced safety-critical events (e.g., crash, near-crash, unintentional lane deviation) in LHTDs by 28–51%. Additionally, reducing fatigue may also reduce stress, which is also associated with risky driving behaviours [22]. While there are truck stops in the major cities in Saskatchewan (e.g., Saskatoon, Regina, Moose Jaw), little is known about whether truck drivers have adequate access to truck stops and rest areas along major routes that lead into the cities. The objectives of this study are twofold: 1) to examine where fatigue-related accidents occur in Saskatchewan; and 2) to assess the perceptions of LHTDs concerning fatigue and access to truck stops and rest areas. The findings from this study may help policy makers and LHTD stakeholders identify optimal areas of intervention (where truck stops/rest areas could be developed) to reduce the risk of crashes.

## Methods

### Protocol

To determine the location of fatigue-related crashes in Saskatchewan, three databases from Saskatchewan Government Insurance (SGI) were retrieved consisting of driver records from commercial motor vehicle (CMV) drivers from 2007 to 2017. The first was a master list of all CMV drivers in the province; the second was a list of all police reported CMV crashes; and the third was a list of all medical conditions that CMV drivers reported to SGI. All data were de-identified by SGI prior to release.

More than 176,000 drivers were included in the master SGI database that included information on date of birth (age), gender, license type and years of CMV experience. All databases were cleaned and filtered to retrieve only Class 1 licences which are specific to commercial heavy and tractor-trailer truck drivers. Overall, there were 86,272 Class 1 truck drivers in the master database. Driver ID numbers were used to link all three databases. Codes were provided by law enforcement officials who responded to the crashes. There were four broad categories of codes consisting of human, vehicle and environmental conditions, respectively, as well as human action. Under human conditions, there were 12 options from which police officers could select, two of which were listed as extreme fatigue (defined as normal physical exhaustion not caused by drugs or a medical condition) and falling asleep. The location of the crash was also documented. As there was overlap of drivers categorized as experiencing extreme fatigue and who had fallen asleep, drivers who were reported having both were categorized as falling asleep for data analysis, as falling asleep at the wheel includes extreme fatigue and is more serious than fatigue alone. The variable “extreme fatigue” includes those who were fatigued without falling asleep.

Using this information, we identified where fatigue and sleep-related crashes occurred and plotted the crash location on a map of Saskatchewan: http://ontheworldmap.com/canada/province/saskatchewan/saskatchewan-highway-map-max.jpg. Highways were examined by routes, geography (east, west, north and south), speed limit, two vs. four-lane highways, radius and whether they intersected with a major city with a truck stop (e.g., Saskatoon or Regina).

Locations of truck stops and rest areas were searched using Google and were cross-listed with “Allstays,” an online directory (https://www.allstays.com/c/truck-stops-saskatchewan.htm) listing the number of parking spaces available, restaurants and amenities (e.g., showers, payphone, internet). A truck stop is defined as being on the highway or close to the highway and is used to rest, eat and refuel [23]. Meanwhile, rest stops are defined as areas nearby a highway (or next to the highway) where drivers can stop to rest and use washrooms [24]. Based on this information, there were 45 truck stops; 38 of them being defined as small [25]. Of the small truck stops, 13 did not have parking spaces, 5 provided no information on the number of parking spaces, 15 had between 2 and 10 parking spaces, and 5 had 12–20 parking spaces. As truck drivers do not plan on stopping at small truck stops [26], we only plotted truck stops with more than 20 parking spaces on the map. Only 7 truck stops had more than 20 parking spaces, with two locations in Saskatoon, two in Regina, and one in Swift Current, Whitewood, and Moosomin, respectively. There were 21 rest stops across Saskatchewan with no rest stops specific for truck drivers. Only three rest stops advertised they had parking available for truck drivers, usually 4–5 parking spaces, along with a restroom and running water.

To gather additional information on fatigue, and access to truck stops and rest areas in Saskatchewan, LHTDs were recruited to participate in a semi-structured interview from seven truck stops in Alberta and Saskatchewan. Truck drivers were approached to participate in an interview by the researchers at each of the truck stops. To be eligible, truck drivers were required to be: 1) Canadian; 2) have a Class 1 driver’s license (or equivalent); and 3) had spent at least one night away from home while delivering a load at the time of recruitment. Participants were asked open-ended questions on driving routes, access to truck stops and rest areas, availability of truck stop/rest area amenities, and required improvements to reduce fatigue among LHTDs. The interview guide is published elsewhere [27]. The interviews took, on average, 30 min to complete; all interviews were audio-recorded for transcription purposes. Sixty-seven participants completed interviews. The mean age of the sample was 53.0 ± 12.9 (range 23–89; 95% were men). Each participant was compensated $10 for their time. This study was approved by the Research Ethics Board at the University of Saskatchewan.

### Analyses

The locations of the crashes were used to create a heat map to show where collisions occurred due to falling asleep, with cooler and hotter colours indicating fewer or more collisions, respectively. Demographic information including age, gender, medical conditions and crashes were described using descriptive statistics (e.g., Mean ± SD or frequency and percent). Group comparisons were performed using either a chi-square or an Analysis of Variance. Correlations, chi-squares or independent t-tests were performed to examine the association between the number of fatigue-related crashes and highway characteristics (e.g., routes, two vs. four-lane highways, length, and whether they have access to a truck stop).

All interviews were transcribed by the Social Sciences Research Laboratories at the University of Saskatchewan. NVivo 11, a qualitative data management tool, was used to analyze all 67 transcripts. Thematic analysis was used to analyze the data which consisted of identifying and recording patterns within the data. Initial codes were developed deductively, based on the research questions, with information separated into comments and recommendations. Subsequent sub-codes were developed by identifying commonalities within the data and organizing information into increasingly specific groups. By grouping common codes together, the final themes were identified. Data was analyzed using two independent coders; one identified the initial codes and sub-codes and the other confirmed their accuracy and developed the final overarching themes.

## Results

Over the 10-year period, there were a total of 201 crashes that were caused by extreme fatigue and/or falling asleep while driving (0.6% of all crashes): 79 crashes involved extreme fatigue and 154 crashes were from falling asleep. There were also 32 cases who crashed that were listed as both extremely fatigued and had fallen asleep at the wheel. Only seven drivers who crashed had a diagnosed medical condition: 5 had diabetes, 1 had a stroke, and 1 had heart problems. Table 1 shows the demographic profile of the sample. There was a significant age difference between drivers who crashed that were extremely fatigued and/or whom fell asleep compared to drivers who remained awake (*F*(2,32,471) = 24.3, *p* < .001). The Scheffe’s Post-Hoc test revealed that the fatigued (Mean = 38.1 ± 13.1 years) and fell asleep (Mean = 38.9 ± 13.6 years) groups were both significantly younger that those who were awake when they crashed (Mean = 45.8 ± 14.4 years, *p* < .001 for both).

**Table 1:** Descriptive variables for drivers who were extremely fatigued, fell asleep, or were awake and crashed

	Extreme Fatigue (*n* = 47)	Fell Asleep (*n* = 154)	Was Awake (*n* = 32,772)
Age (years)	38.1 ± 13.1	38.9 ± 13.6	45.8 ± 14.4
Gender (% male)	93.6%	100%	96.9%
Years Licensed	6.5 ± 5.9	7.6 ± 6.5	10.5 ± 7.7
Medical condition affecting sleep	2.1%	3.9%	2.6%

There was a significant relationship between years of experience and crashes (*F*(2,32,471) = 17.6, *p* < .001). Drivers who crashed while awake had more experience than both drivers who crashed due to extreme fatigue (Mean = 10.5 ± 7.7 years vs. Mean = 6.5 ± 5.9 years, *p* = .002) and drivers who crashed due to falling asleep (Mean = 10.5 ± 7.7 years vs. Mean = 7.6 ± 6.5 years, *p* < .001). There were no differences in working experience between drivers who were fatigued and crashed and those who fell asleep and crashed. There were no gender differences across groups nor were there any significant associations between medical conditions and fatigue.

**Fig. 1:**
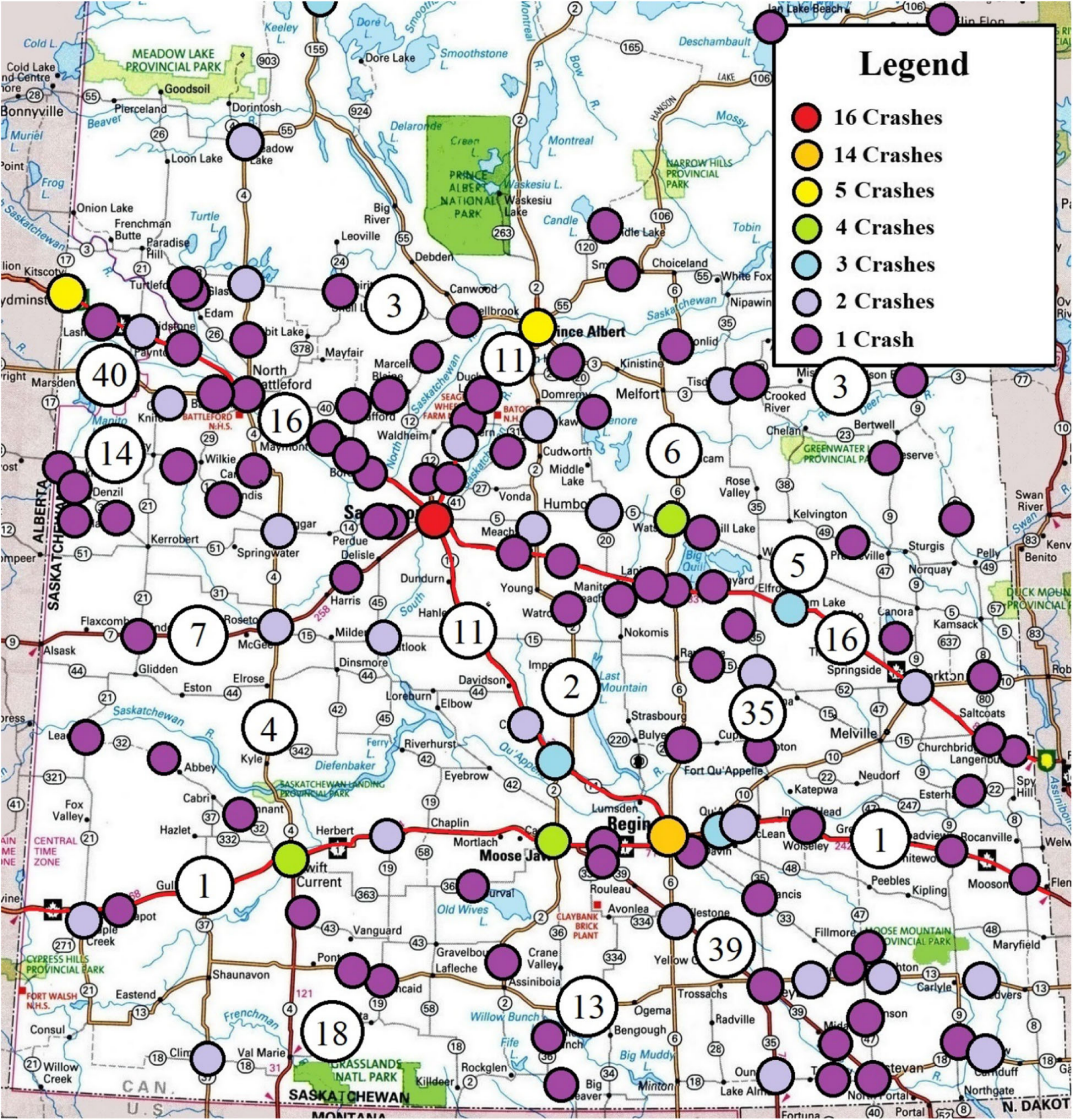
All crashes related to extreme fatigue and falling asleep behind the wheel in Saskatchewan over a 10-year period

Figure 1 shows where crashes occurred across Saskatchewan over a ten-year period due to drivers being either extremely fatigued or falling asleep. The largest concentrations of crashes were located in major centres: Saskatoon (*n* = 16) and Regina (*n* = 14). These two locations account for 13.6% of all crashes from falling asleep. The majority of the crashes outside those two cities are located on highways throughout the province.

Table 2 shows the highway number, location and their characteristics (e.g., number of lanes, speed, length, access to truck stops) where fatigue-related crashes occurred. The number of crashes was significantly associated with highway length (*F*(10,18) = 4.17, *p* = .027), although there were no significant association with number of crashes and the number of lanes (*t*(17) = − 0.87, *p* = .39) or whether or not there was access to rest areas (*t*(17) = 0.43, *p* = .68). However, when the number of crashes were associated with highway length on 2 and 4-lane highways separately, there was a significant association between the number of crashes and highway length on 2-lane highways (*F*(8,12) = 14.4, *p* = .01). No relationship was found between number of crashes and highway length on 4-lane highways (*F*(4,5) = 1.59, *p* = .53).

**Table 2:** Characteristics of all Saskatchewan Highways

Highway #	Location	Truck Stops along the highway	# Truck Stops	# Lanes	Length (km)	# Crashes
1	West of Regina	Yes	1	4, divided	400	14
1	East of Regina	Yes	1	4, divided	235	9
11	Regina to Saskatoon	Yes	2	4, divided	260	5
11	Saskatoon to Prince Albert	Yes	1	4, divided	140	5
16	West of Saskatoon	Yes	1	4, divided	275	13
16	East of Saskatoon	Yes	1	4, divided	410	12
2	Prince Albert to Rockglen	No	0	2	500	12
3	West of Prince Albert	No	0	2	310	5
3	East of Prince Albert	No	0	2	300	5
4	Meadow Lake to Monchy	No	0	2	610	16
5	East of Saskatoon	Yes	1	2	380	7
6	North of Regina	Yes	1	2	350	7
7	West of Saskatoon	Yes	1	2	260	4
13	Govenlock to Antler	No	0	2	650	10
14	West of Saskatoon	Yes	1	2	250	7
18	Robsart to Gainsborough	No	0	2	690	10
35	Nipawin to Oungre	No	0	2	500	9
39	South of Regina	No	0	2	275	6
40	Shellbrook to Marsden	No	0	2	280	8

The association between number of crashes and whether there were truck stops/rest areas on 2-lane highways were examined separately. Additionally, the association between the number of crashes and whether there were one or two truck stops on 4-lane highways were assessed. The findings showed no significant association between the number of crashes and availability of truck stops/rest areas on 2-lane (*t*(11) = 1.46, *p* = .17) or 4-lane highways (*t*(4) = 1.40, *p* = .23). However, when the number of crashes were controlled for by length of highway, the number of crashes were significantly lower on 4-lane highways with two truck stops/rest areas compared to one or no truck stops/rest areas (*p* < .05). No differences on 2-lane highways emerged.

Overall, truck drivers who participated in the interview overwhelming mentioned that there is a shortage of trucks stops and rest areas in the Western Canadian Provinces, as well as Canada generally. Additionally, there is insufficient parking available at the truck stops available. Due to these parking issues, LHTDs said they will either choose to drive while fatigued or park in unsafe areas. When asked how far apart and what should be provided at truck stops and rest areas, LHTDs stated that they should be 50–200 km apart and should at least have a bathroom and running water.

### Access to truck stops and rest areas

Participants were asked whether they were satisfied with the current amount of truck stops and rest areas available to them. Almost all of the participants (91%) stated that there are not enough truck stops and/or rest areas in Canada. Many participants also highlighted Saskatchewan as being particularly problematic for having insufficient truck stops and/or rest areas compared to other Canadian provinces. Participants reported the inability to find parking along highways/driving routes to be quite stressful.

*Even those rest stops on, or turn offs, really there isn’t enough of them compared to the amount of trucks on the road. –* Participant 16

*Yeah rest stops here in Canada are garbage. In Saskatchewan there’s only like two rest stops that trucks are allowed to go in. –* Participant 21

*Think the biggest problem Canada has is the laws say you have to break every four hours. There’s not a rest stop every four hours so you might as well throw that right out the window.* – Participant 49

*My biggest frustration when I’m on the road is not having enough rest areas. –* Participant 52

### Parking availability

Parking was the primary concern of our participants in relation to insufficient truck stops and rest areas. Almost all participants (96%) stated that there was not enough parking, even when there were truck stops or rest areas.

*Every truck stop should have triple the parking that it has. –* Participant 7

*You’ve got lack of parking. It makes it all stressful. –* Participant 24

*The biggest thing I have found with truck stops is there is no place for us to park, that’s the big thing. It drives me crazy. –* Participant 36

### Driving while fatigued

Many drivers reported often being forced to drive to the next location in hopes that there are spots to park. In some cases, participants reported to continue driving despite being fatigued. This is especially a concern for those that start driving in the morning and finish their shift at night.

*If you’re tired and you’re looking to pull-in and oh, it’s all full, now you’ve got to make the decision: do I keep pushing it or do I do ‘til the next one? And if you don’t know when the next one is, then you’re going to start pushing the envelope top the edge, and you’re already tired; that’s why you are looking for that rest area. –* Participant 15

*Just a place to pull over for the night, you get to the truck stops, take a look outside it’s loaded. It’s filled up by seven o’clock, you can’t get in and then try and get out in the morning when you’re blocked in.* – Participant 19

*Oh sure, yeah I come here quite often but if you don’t get here early you’re not getting a parking spot. Then you’re parking down at the scale or somewhere in the city, somewhere else.* – Participant 24

*Lack of places that a guy can’t stop for a bathroom break or for coffee break. Anything like that eh. And sleeping at night, that’s… Depending on where you are, I just run, now I just run Alberta, Saskatchewan, Manitoba. –* Participant 91

### Impact of hours of service regulations

The lack of truck stops and rest areas were also a concern due to the hours of service regulations. Many drivers reported parking on the side of the road if their hours of service requirements have been met for the day and there were no truck stops/rest areas. This was noted as being stressful as many drivers then park in areas where safety concerns may exist (e.g., side of the highway; unlit and quiet road). For example, some participants reported needing to leave their trucks and walk a distance to access food and other amenities, and during that time, their trucks were either burglarized or had been attempted to be burglarized. Participants noted poor lighting, traffic, inability to sleep due to noise, and being in an unsecure area.

*When you’re goin’ down the road, and if they came out with e-log in Canada, where you gonna stop alongside the highway when there’s no roadside turn outs? –* Participant 11

*And you get your hours of service, you just wait, you wait till the hours of service and the e-log becomes law, you’re gonna have trucks parked all over the god damn place. All over the place. –* Participant 24

*The only shortfall I got is not enough safe parking anywhere in any of the provinces of Canada. Now with the electronic logs, everybody is fighting to get to the next truck stop so they can find a safe place to park and most of us are being stuck out on the side of the road. –* Participant 30

*There is very few rest stops… I’ve actually parked on the side of the highway, put my four ways on, lights out, put the four ways on, left the truck running. So you still gotta get your sleep. –* Participant 89

### Recommendations for improvement: amenities

The participants also provided input on the current truck stops/rest areas, as well as what they would want to see in newly developed rest areas. More than half of the participants reported that all truck stops and rest areas should have at least a bathroom and, ideally, running water. Even if truck stops have access to bathrooms and food, many participants noted that the lack of hygiene is a concern. Many participants also wanted showers at the truck stops.

*A washroom facility, a garbage can, a pull over, you know, garbage can recycle bin side by each where you can pull over and stop for an hour or do a walkabout on your like load securement, stuff like this. –* Participant 15

*They don’t have enough areas and if they do have areas, there’s no facilities, nobody wants to maintain facilities. So it’s fine and dandy to build all these places you can pullover but if they don’t even put up a bathroom or something like that then what is the point? –* Participant 25

*Some places don’t even supply towels so you have to have your own and a lot of the truck stop showers are filthy. They might rinse them out after every person but you can see mold that has been there, like black mold crawling up the walls and all over for years and years. –* Participant 41

*One thing I want in a rest stop is at least a washroom. They need at least a washroom. –* Participant 42

*I noticed if you south of Highway 1, anywhere in Western Canada, you’d be lucky if there’s a shower with the truck stop. Usually it’s just four pumps in the middle of nowhere. –* Participant 49

*The majority of the truck stops [in the US] are cleaner and better maintained than what we have here in Canada. Our truck stops are not as well maintained, not as clean. The standards are definitely lower up here. That’s what the industry could learn. –* Participant 86

### Recommendations for improvement: distance between truck stops and rest areas

Participants also provided information on how far apart truck stops and rest areas should be. Participants reported that the ideal distance between truck stops/rest areas should be between 50 and 200 km or between 1 to 4 h apart. The participants noted that this would provide more parking spots along routes resulting in reduced stress and fatigue.

*I would say within a couple hours apart, I would say, be good. Like places you go there’s one every hour. Other places you go there’s nothing for five, six hours. –* Participant 7

*Yeah, you know maybe every 50 kilometers would be good. I don’t know, sometimes there’s no rest stop for hundreds of kilometers. –* Participant 14

*I would say every hour maybe, hour and a half max. Because if one is full then you don’t have so far to go and you know another hour and a half or an hour, you can pull into another one. –* Participant 15

## Discussion

Over the 10-year period, there were 201 crashes related to fatigue (on average 20 crashes per year) in Saskatchewan alone. Younger LHTDs (in their 30’s) are more likely to suffer from fatigue-related crashes than older LHTDs. Younger CMV drivers may not receive training in dealing with fatigue, may not perceive driving while fatigued to be risky [28] or they lack experience in dealing with fatigue. Another reason could be lack of access to rest areas and truck stops along major highways.

It is well known that LHTDs work long hours and often report being fatigued [3–5]. Prior studies have reported LHTDs receiving an average of 6 hours of sleep per day [29], which can lead to poorer driving performance and an increased crash risk [1, 5]. Our participants mentioned the lack of available rest areas/truck stops along highways and recommended that there should be rest areas and/or truck stops every 50–200 km or roughly 2 hours apart. Distances between cities often go beyond 300–400 km and many drivers reported being fatigued and stressed over the inability to stop and rest. This is consistent with our findings showing that highway routes ranges from 140 to 690 km in length often without access to any rest areas/truck stops.

The number of fatigue-related crashes was highest in the major cities (e.g., Saskatoon and Regina). This trend could be due to various reasons, including LHTDs being unable to rest prior to arriving in major cities, greater cognitive demands, traffic density and volume. However, the rate of fatigue-related crashes was lower on the divided four-lane highway between Regina and Saskatoon compared to similar highways going east or west from those two cities. Our findings show that fatigue-related crashes on divided four-lane highways are reduced on highways where there is more than one truck stop consistent with McArthur and colleagues [30] who found a direct relationship between greater access to truck stops and a lower number of fatigue-related crashes in the U.S.

**Fig. 2:**
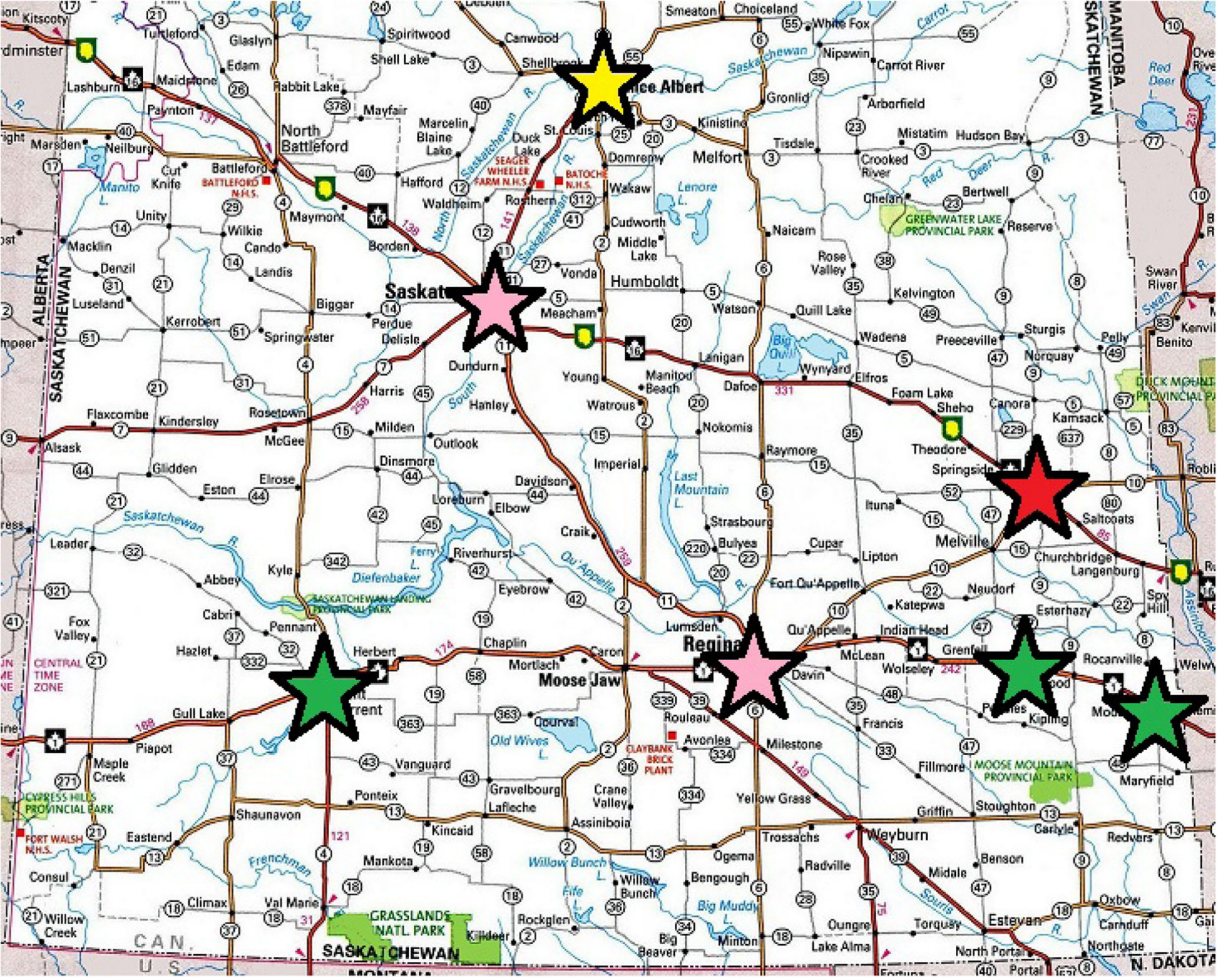
Locations for recommended truck stops

Our findings show that there are fewer crashes when truck stops/rest areas are within 250 km of each other along highway routes. Having truck stops/rest areas 250–300 apart (or 4 h approximately) was considered adequate by our participants and may lead to a reduction in fatigue and fatigue-related crashes. Our findings, based on the location of crashes and participant recommendations, suggest possibilities for the development and expansion of truck stops and/or rest areas in the Saskatchewan cities of Yorkton, and Prince Albert (see Fig. 2). While there are truck stops in both cities, they have limited parking options and amenities for LHTDs. Either the development or expansion of these rest areas would reduce travel-related fatigue and provide access to truck stops/rest areas that are at least 4 hours apart between the major cities of Saskatchewan (Regina and Saskatoon) and Alberta and Manitoba. Other recommended locations are Moosomin, Swift Current and Whitewood which have only recently been expanded to include more parking spaces and amenities (e.g., showers, restaurant). This is consistent with the needs of the participants stating the desire for adequate lighting, bathrooms, running water and showers.

While the recommendation of developing truck stops/rest areas is easy in principle, it requires the coordinated efforts between truck drivers, the public and private sector to balance the costs associated with building and maintaining the truck stops/rest areas. Additionally, Montufar and colleagues [18] suggests developing real time software applications that can provide real time data on parking options, as well as developing a nationwide inventory of parking facilities (and their amenities) so drivers can plan their routes.

While this study provides new data related to the location of truck stops and crashes, there are a few limitations. Convenience sampling was utilized to identify participants for the semi-structured interviews; consequently, the participants may not have been representative of all LHTDs. However, convenience sampling is typically used in qualitative studies and given the large number of interviews performed, it is likely that a wide range of perspectives were gathered. Although we could determine the number of crashes and the location of the crashes, we could not determine the driving conditions (e.g., highway volume or traffic, bad weather) at the time of the crash. We also lacked information on the time of day the accident occurred and could not determine whether crashes occurred in peak traffic, at night or during the daytime. We also did not have data on how long the driver was driving prior to the crash nor any information on their decision to drive particular routes or highways. For this reason, it is difficult to discern whether particular highways can lead to an increased risk of crashes or whether it is simply a product of opportunity (e.g., more drivers on highways leads to more opportunity to crash). Additionally, we could not determine traffic volume on the respective roadways. However, these limitations can be potentially addressed using data from the Canadian Naturalistic Driving Study (CNDS) that prospectively tracked truck drivers over 2 years [31]. The CNDS collected information on truck driver’s driving exposure and patterns using GPS devices, on-board diagnostic devices, various cameras and sensors, as well as eye monitors. Using such data could confirm whether LHTDs are indeed involved in fatigue-related crashes based on eye movements, length of time driving, traffic density and volume, as well as road and weather conditions.

## Conclusions

The study results support the existence of an association between fatigue-related crashes and a lack of truck stops and rest areas. The lack of truck stops and rest areas was reinforced by the perceptions of the LHTDs we interviewed. Given the large number of truck drivers on the road and the limited areas for rest, vested stakeholders are urged to consider developing additional truck stops and rest areas with essential amenities to reduce the risk of fatigue-related crashes. Mobile or web-based applications are needed to provide real-time information on parking availability along highway/freeway routes for LHTD and future studies should assess the impact of newly implemented truck and rest stops on driver fatigue and fatigue-related crashes in LHTD.

## Data Availability

The datasets pertaining to SGI (CMV drivers) are publicly available and can be provided from the corresponding author on request. With respect to the interview data, the data that support the findings of this study are not available for another 2 years, based on the licensing agreement between the corresponding author and the Alberta Ministry of Labour. However, data requests can be made to the corresponding author upon reasonable request and with permission from the Alberta Ministry of Labour.

## Abbreviations

LHTD: Long-haul truck drivers
CMV: Commercial motor vehicle
SGI: Saskatchewan Government Insurance
